# CT-Based Radiomics for Prediction of Molecular Markers in Clear Cell Renal Cell Carcinoma: A Comprehensive Review

**DOI:** 10.3390/medicina62071349

**Published:** 2026-07-12

**Authors:** Ekaterini Boukali, Petros Koumpis, Eleni Romeo, Eyrysthenis Vartholomatos, George A. Alexiou, Maria I. Argyropoulou, Athina C. Tsili

**Affiliations:** 1 Department of Clinical Radiology, University Hospital of Ioannina, University Campus, 45110 Ioannina, Greece; katboukali@gmail.com (E.B.); petroskoumpis@gmail.com (P.K.); eyrys.varth@gmail.com (E.V.); 2 Department of Neurosurgery, Faculty of Medicine, School of Health Sciences, University of Ioannina, University Campus, 45110 Ioannina, Greece; e.romeo@uoi.gr (E.R.); galexiou@uoi.gr (G.A.A.); 3 Department of Clinical Radiology, Faculty of Medicine, School of Health Sciences, University of Ioannina, University Campus, 45110 Ioannina, Greece; margyrop@uoi.gr

**Keywords:** renal cell carcinoma, clear cell renal carcinoma, multidetector computed tomography, radiomics, machine learning, tumor biomarkers

## Abstract

*Background and Objectives:* Clear cell renal cell carcinoma (ccRCC) demonstrates substantial molecular and clinical heterogeneity, limiting the prognostic accuracy of conventional staging system and complicating treatment selection. CT-based radiomics and radiogenomics have emerged as promising non-invasive approaches for predicting molecular biomarkers. This review aimed to evaluate the current evidence regarding CT-based radiogenomics for the prediction of molecular markers in ccRCC, with emphasis on methodological approaches, predictive performance, and clinical applicability. *Materials and Methods:* A comprehensive literature search of PubMed/MEDLINE, Scopus, and Cochrane Library databases was performed for original studies published between January 2012 and December 2025. Eligible studies included patients with histopathologically confirmed ccRCC, performed CT-based radiomics feature extraction, and investigated molecular or genetic biomarkers using machine learning (ML) methods. Data regarding CT acquisition phase, segmentation strategy, radiomics features, ML algorithms, investigated biomarkers, and model performance metrics were extracted. *Results and Discussion:* Twenty-five retrospective studies were included. CT-based radiomics demonstrated promising performance in predicting gene mutations, including Von Hippel–Lindau (VHL), Polybromo 1 (PBRM1), BRCA1-associated protein 1 (BAP1), SET domain containing 2 (SETD2), and Lysine demethylase 5C (KDM5C), with reported area under the curve (AUC) values reaching 0.987. Radiogenomic models also showed utility in assessing hypoxia-related pathways, lipid metabolism signatures, programmed cell death profiles, immune-related markers, and tumor microenvironment characteristics, including programmed death-ligand 1 (PD-L1), Cluster of Differentiation 68 (CD68+) tumor-associated macrophages (TAMs), Cytotoxic T-Lymphocyte–Associated Protein 4 (CTLA-4), Forkhead Box P3 (FOXP3), and Ki-67 proliferation index. Predictive performance varied across biomarkers, with AUCs generally ranging from 0.68 to 0.91. Random Forest (RF), Logistic Regression (LR), Support Vector Machine (SVM), Adaptive Boosting (AdaBoost), and Gradient Boosting algorithms were most commonly applied. *Conclusions:* CT-based radiogenomics represents a promising non-invasive tool for molecular characterization and risk stratification in ccRCC. Standardized multicenter prospective studies, methodological homogeneity, and external validation are required before routine clinical implementation.

## 1. Introduction

Renal cell carcinoma (RCC) is the most common kidney malignancy in adults, accounting for approximately 3% of all cancers worldwide [1]. Over the past two decades, its global incidence has increased steadily by approximately 2% annually, with projections estimating 745,791 new cases and 304,861 deaths by 2050 [2].

Clear cell renal cell carcinoma (ccRCC), the most prevalent histological subtype, is characterized by significant clinical and molecular heterogeneity, which complicates treatment decision-making and limits the predictive accuracy of conventional prognostic systems such as Tumor-Node-Metastasis (TNM) staging [1,2,3]. Although surgical resection remains the standard of care for localized disease, approximately 20–30% of patients develop recurrence or metastatic progression, highlighting the need for improved preoperative risk stratification [3,4,5,6,7].

In advanced disease, targeted therapies and immune checkpoint inhibitors (ICIs) have improved clinical outcomes; however, treatment responses remain highly variable. This variability reflects the complexity of the tumor microenvironment (TME) and the underlying molecular landscape of ccRCC [8,9,10,11,12]. Several molecular biomarkers, including hypoxia-inducible factors (HIFs), vascular endothelial growth factor (VEGF), programmed death-ligand 1 (PD-L1), and Ki-67, are associated with key biological processes such as hypoxia signaling, angiogenesis, proliferation, and immune evasion, and have demonstrated prognostic potential. In addition, gene expression profiles, microRNAs, and epigenetic alterations further capture tumor heterogeneity [8,9,13,14]. However, their clinical utility remains limited due to inconsistent validation, tumor heterogeneity, and reliance on invasive tissue sampling. Therefore, there is a critical need for non-invasive, reproducible biomarkers to improve prognostic assessment and guide personalized treatment strategies.

Radiomics has emerged as a promising non-invasive approach that enables the extraction of high-dimensional quantitative features from medical images. As contrast-enhanced computed tomography (CECT) is routinely used for the diagnosis, staging, and follow-up of RCC, it represents a valuable source of imaging data. Radiomic features describing tumor shape, intensity, and texture can capture intratumoral heterogeneity beyond visual assessment [8,9]. Radiogenomics extends this concept by linking imaging features with underlying molecular and genetic characteristics, potentially enabling non-invasive prediction of tumor biology.

The integration of machine learning (ML) techniques has further enhanced the performance of radiomics by enabling the identification of complex, non-linear relationships between imaging features and clinical or molecular outcomes [8,9,15,16,17]. Despite these advances, several challenges limit clinical implementation, including the lack of standardized imaging protocols, variability in feature extraction, small and heterogeneous study cohorts, limited external validation, and concerns regarding reproducibility.

Given the rapid development of this field, a comprehensive evaluation of current evidence is needed. This review aims to assess the role of CT-based radiogenomics in the non-invasive prediction of molecular markers in ccRCC, with a focus on methodological approaches, clinical applicability, and future perspectives in precision oncology.

## 2. Materials and Methods

### 2.1. Literature Search Strategy

A comprehensive literature search was conducted in three electronic databases (PubMed/MEDLINE, Scopus, and Cochrane Library) to identify relevant studies published between January 2012 and 31 December 2025.

The search strategy was developed using a combination of Medical Subject Headings (MeSH) and free-text terms, including: “renal cancer” (OR) “renal cell renal cell carcinoma” (OR) “clear cell renal cell carcinoma” (OR) “computed tomography” (OR) “CT” (OR) “radiomics” (OR) “radiogenomics” (OR) “molecular markers”. The search queries were appropriately adapted for each database.

After the removal of duplicate records, three independent reviewers (PK, ER and EV) screened the titles and abstracts to exclude irrelevant studies. Full-text versions of the remaining studies were then assessed for eligibility according to predefined inclusion and exclusion criteria. Any discrepancies during the selection process were resolved through discussion and consensus with the corresponding author.

### 2.2. Selection Criteria

The inclusion criteria were as follows: studies involving patients with histopathologically confirmed ccRCC; studies performing radiomics feature extraction from CT images; and studies providing sufficient methodological details for data extraction, including CT acquisition phase, segmentation approach, radiomic features extracted, and ML models. Additionally, information on the investigated molecular or genetic biomarkers was collected, along with key study outcomes such as model performance metrics, including the area under the curve (AUC). For performance metrics, external validation results were preferentially extracted when both internal and external validation data were available. When studies reported multiple models or outcomes, those with the best reported performance were selected for extraction.

Studies were excluded according to the following criteria: (1) non-original articles, such as reviews, editorials, case reports or case series, conference abstracts, and book chapters; (2) publications not written in English; (3) studies involving non-solid or secondary renal tumors or uncommon RCC histological subtypes; (4) studies focusing on pediatric patients; and (5) studies reporting clinically irrelevant outcomes.

### 2.3. Descriptive Synthesis of Model Performance

For descriptive purposes, reported AUC values of radiomics signature models were extracted. Clinical models and radiomics nomograms were recorded separately when available. When multiple molecular outcomes were reported within the same study and biomarker category, all values were retained in the detailed table. For the conservative study-level summary and visualization, each study contributed once within each biomarker category; when multiple outcomes were reported within the same category, the median AUC was used to avoid overrepresentation of studies reporting multiple endpoints. Descriptive plots were generated to visually summarize the distribution of study-level AUC values across biomarker categories.

Because of substantial heterogeneity across CT protocols, segmentation approaches, radiomics pipelines, molecular targets, and ML models, the findings were synthesized narratively rather than quantitatively. These plots were intended only to support narrative interpretation and were not considered a quantitative synthesis or meta-analysis.

## 3. Results

An initial comprehensive search of three electronic databases—PubMed/MEDLINE (*n* = 252), Cochrane Library (*n* = 13), and Scopus (*n* = 368)—identified a total of 633 records. Following the removal of 231 duplicate records, 402 unique citations remained and underwent title and abstract screening. During this stage, 209 records were excluded, resulting in 193 articles deemed potentially eligible for full-text retrieval. All 193 full-text articles were successfully retrieved and assessed for eligibility. Ultimately, 25 retrospective studies (*n* = 7274) fulfilled the predefined inclusion criteria and were included in the review [18,19,20,21,22,23,24,25,26,27,28,29,30,31,32,33,34,35,36,37,38,39,40,41,42].

**Figure 1:**
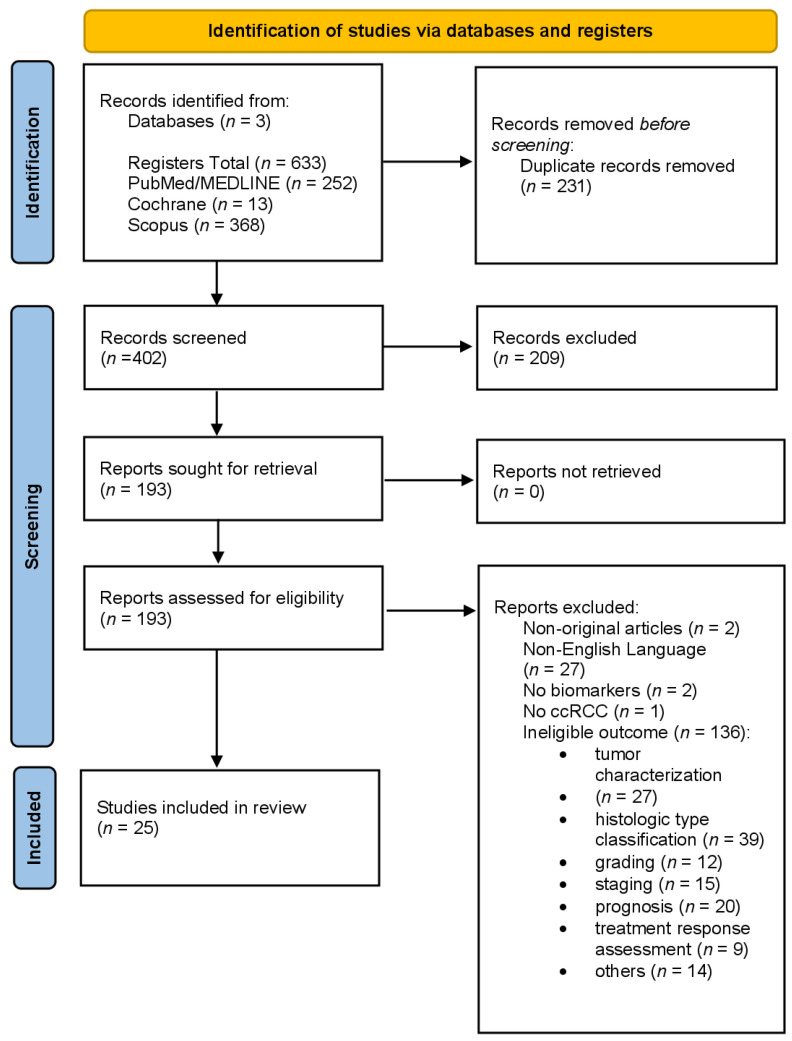
Study selection flowchart.

The search findings are summarized in the flow chart presented in Figure 1. The baseline characteristics and CT-based radiomics pipeline characteristics of the studies investigating molecular marker prediction in patients with ccRCC are summarized in Table 1 and Table 2.

Among the included studies, nine were multicenter studies [18,19,21,22,26,31,32,35,36] and 16 were single-center studies [20,23,24,25,27,28,29,30,33,34,37,38,39,40,41,42]; 16 of these studies used data from The Cancer Genome Atlas Kidney Renal Clear Cell Carcinoma (TCGA-KIRC), and The Cancer Imaging Archive (TCIA) public databases [23,24,25,26,28,29,30,34,35,36,37,38,39,40,41,42]. In all studies, radiomics signature models were used to predict molecular or biological biomarkers. Of these, three studies additionally evaluated clinical models and radiomics nomograms [26,34,39].

Among the 25 studies, 11 included external validation [18,19,21,22,23,24,25,26,31,36,42], and 14 relied on internal validation or cross-validation within the same cohort [20,27,28,29,30,32,33,34,35,37,38,39,40,41].

**Figure 2:**
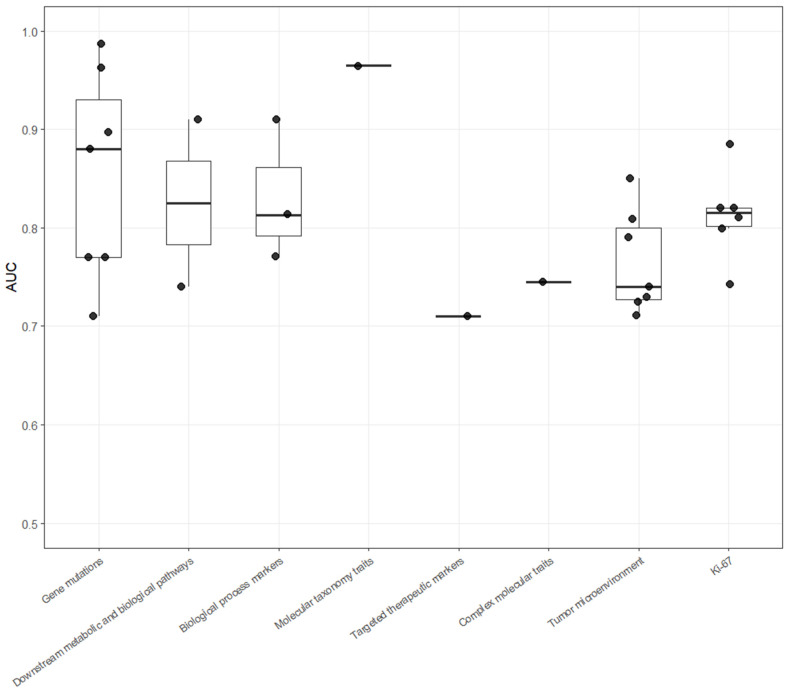
Study-level distribution of reported AUC values for CT-based radiomics according to molecular marker category. Each point represents one study within a biomarker category. When a study reported multiple outcomes within the same category, the median AUC was used to avoid overrepresentation. Boxplots show the median and the interquartile range.

The resulting AUC distribution by biomarker category is summarized in Table 3 and visually presented in Figure 2.

## 4. Discussion

### 4.1. Gene Mutations

Advances in genomic research have established that gene mutations are the primary architects of ccRCC pathophysiology, serving as the essential “blueprint” for tumor progression and therapeutic vulnerability [9]. These genetic alterations drive malignancy by directly reprogramming cellular proliferation, metabolism, and apoptosis, while simultaneously disrupting signaling networks that facilitate invasive behavior and drug resistance. Therefore, these mutations not only dictate the tumor’s biological behavior but also define the specific molecular targets for modern precision medicine. Key gene mutations identified in ccRCC include the Von Hippel-Lindau (VHL) tumor suppressor, Polybromo 1 (PBRM1), BRCA1-associated protein 1 (BAP1), SET domain containing 2 (SETD2), and Lysine demethylase 5C (KDM5C), most of which are located on the short arm of chromosome 3 (3p) [9,14].

Existing techniques for assessing gene mutations in ccRCC have several important limitations [9,36,41]. Tissue biopsy and sequencing, while considered the reference standard, are invasive, costly, and not always feasible for routine or repeated use. Moreover, biopsy samples are typically small and may fail to capture the marked intratumoral heterogeneity characteristic of ccRCC, leading to incomplete or potentially misleading representations of the tumor’s mutational landscape. Comprehensive genomic profiling is more reliably obtained from surgically resected specimens, but this approach is not scalable, cannot be applied preoperatively, and is impractical for longitudinal monitoring. Consequently, there is a clear need for non-invasive, cost-effective methods capable of capturing the full spatial and temporal complexity of tumor genetics.

#### 4.1.1. VHL

The biallelic inactivation of the *VHL* tumor suppressor gene serves as the critical “truncal” mutation for ccRCC tumorigenesis, occurring in 50–90% of cases and frequently correlating with chromosome 3p deletion [35,41,43]. This loss of function disrupts the cell’s ability to adapt to hypoxia by preventing the targeted degradation of HIFs (HIF-1α and HIF-2α). The resulting stabilization of HIF transcription factors triggers a constitutive pseudo-hypoxic response, initiating a cascade of metabolic reprogramming and pathological angiogenesis. This process drives the overproduction of pro-angiogenic and metabolic targets, such as VEGF and Glucose Transporter 1 (GLUT1), which facilitate the hyper-vascularization and aerobic glycolysis characteristic of the clear cell phenotype [35,41,43]. While this pathway has provided the biological rationale for successful targeted therapies, such as the VEGF pathway blockers sorafenib and axitinib, comprehensive meta-analyses indicate that the *VHL* mutation itself lacks significant independent prognostic or predictive value in clinical practice [44].

#### 4.1.2. PBRM1

The *PBRM1* mutation represents the second most common genetic alteration in ccRCC and is generally associated with a more favorable prognosis and increased recurrence-free survival (RFS) [30,35,40,41,45]. As a key subunit of the SWItch/Sucrose Non-Fermentable (SWI/SNF) chromatin-remodeling complex, PBRM1 helps regulate cellular proliferation and chromosomal stability; its loss of function is linked to specific therapeutic sensitivities, particularly showing an enhanced benefit from Anti-Programmed Cell Death Protein 1 (anti-PD1) immunotherapy and VEGF-targeted therapies [40,41,46,47,48].

#### 4.1.3. BAP1

The *BAP1* mutation, occurring in approximately 10–15% of ccRCC cases, serves as a critical determinant of tumor aggressiveness and a primary biomarker for personalized precision therapy [35,37,38,41,42,49,50,51,52,53,54,55,56]. The loss of this nuclear deubiquitinase is strongly correlated with advanced clinicopathological features, including high nuclear grades, coagulative tumor necrosis, and metastatic disease at presentation, which collectively result in significantly shorter overall survival (OS) and progression-free survival (PFS) [35,41,42,50,51,52,53,54,55,56,57]. Mechanistically, BAP1 deficiency is often associated with the activation of the mammalian target of rapamycin (mTOR) pathway, which may contribute to the poor clinical outcomes and diminished response to targeted therapies, such as everolimus, observed in these patients. Despite its association with a poor prognosis, BAP1 status offers expanding opportunities for therapeutic intervention. While ccRCC is traditionally radioresistant, recent evidence suggests that *BAP1* loss may sensitize tumor cells to radiation therapy and poly (ADP-ribose) polymerase inhibitors. Furthermore, the functional relationship between BAP1 and ubiquitin ligases has paved the way for novel strategies, including the clinical investigation of histone deacetylase inhibitors for refractory metastatic RCC [37,51,52,53,54].

#### 4.1.4. SETD2

In ccRCC, *SETD2* mutations occur in approximately 10–15% of cases and are strongly associated with advanced tumor stage, high nuclear grades, and a significantly higher propensity for metastasis. Clinical data indicate that SETD2 deficiency serves as an independent marker of poor prognosis, correlating with reduced OS and PFS. This aggressive phenotype is increasingly being addressed through precision medicine; recent evidence suggests that *SETD2*-mutant cells exhibit a unique vulnerability to Protein Kinase B (AKT) and Phosphoinositide 3-Kinase Beta (PI3Kβ) pathway inhibitors, thereby offering a strategic therapeutic window for patients who typically show poor responses to conventional anti-angiogenic therapies [30,35,51,53].

#### 4.1.5. KDM5C

KDM5C, mutated in 7–10% of ccRCC cases, triggers significant chromatin disruption and genomic rearrangement upon its loss, fostering an environment prone to tumorigenesis. Clinically, *KDM5C* mutations serve as a marker for poor prognosis and advanced disease. This epigenetic vulnerability has led to the development of a novel class of KDM5 demethylase inhibitors, which aim to correct the aberrant transcriptional programs driven by KDM5C deficiency [30,35,57].

#### 4.1.6. Radiomics Prediction of Mutational Status

Recent advancements in radiogenomics have demonstrated that CT-based radiomics can serve as a high-fidelity surrogate for identifying the specific genetic architecture of ccRCC [30,35,37,38,40,41,42]. By utilizing an ML algorithm—specifically Random Forest (RF)—researchers have demonstrated that quantitative radiomics features extracted from CECT scans can accurately predict critical truncal and secondary mutations. The study achieved high discriminatory power for VHL (AUC = 0.971), PBRM1 (AUC = 0.972), BAP1 (AUC = 0.955), and SETD2 (AUC = 0.949) [35]. A manual three-dimensional (3D) segmentation technique was applied, extracting 107 radiomics features (shape, histogram, and texture]. Furthermore, the study reported that the fusion of radiomic signatures with genetic mutational status improved the survival prediction in ccRCC patients [35].

Utilizing CT data from the corticomedullary phase (CMP), researchers achieved an AUC exceeding 0.85 for the *VHL*, *PBRM1*, and *BAP1* gene mutations [41]. Technically, the prediction of *VHL* mutations was driven by first-order intensity features, specifically Mean and Kurtosis; a lower Kurtosis—characterized by an elongated histogram tail representing a broader distribution of tumor tissue intensity—was indicative of a higher mutational probability. In contrast, the identification of *PBRM1* necessitated a high-dimensional approach, frequently incorporating a combination of nine histogram and textural features. For the more aggressive *BAP1* mutation, the radiomic signature was defined by increased structural disorder. This lack of voxel-to-neighbor uniformity was captured by increased Variance—reflecting significant intensity fluctuations between adjacent voxels—and decreased Homogeneity, which characterizes a non-uniform or focal intensity distribution [41].

In a retrospective evaluation, researchers utilized high-dimensional radiomics features extracted from CMP scans to train complex classifiers for predicting *PBRM1* mutation status. Following the extraction of 828 features—encompassing histogram, texture, and transform-based metrics from manual two-dimensional (2D) segmentations of renal tumors—the study compared the efficacy of Artificial Neural Network (ANN) and RF algorithms. The results demonstrated that the RF classifier, utilizing only five optimized features, significantly outperformed the ANN, achieving a predictive accuracy of 95.0% and a superior AUC of 0.987 [30].

Interestingly, unenhanced CT (UECT) texture analysis has proven to be a feasible and highly accurate method for the non-invasive prediction of *BAP1* mutations. In a dedicated study, researchers extracted 744 features—encompassing histogram, texture, and transform-based metrics—from UECT scans via manual 2D segmentation. By utilizing a RF classifier, they demonstrated high diagnostic performance, achieving an AUC of 0.897 and an overall classification accuracy of 84.6% [37]. These findings are particularly significant as they suggest that genomic insights can be gleaned even from non-contrast CT, providing a critical diagnostic alternative when contrast agents are contraindicated.

By extracting over 2800 robust radiomic features across fifteen texture families and employing ML algorithms such as RF and Adaptive Boosting (AdaBoost), investigators found that while initial biomarker discrimination was relatively weak (AUC = 0.60–0.68), stratification by stage (I/II vs. III/IV) and grade (1/2 vs. 3/4) yielded superior results. Under these stratified conditions, the radiogenomic models achieved acceptable to excellent predictive performance for KDM5C, SETD2, and PBRM1 mutation status, with AUC values ranging from 0.70 to 0.86 [30].

### 4.2. Downstream Metabolic and Biological Pathways

The expression status of hypoxia-related genes, including survival-linked markers such as IFT57, PABPN1, RNF10, RNF19B, and UBE2T, serves as a critical determinant of patient prognosis and therapeutic response [36,58,59]. Recent evidence suggests that CT radiomics can serve as a robust, non-invasive “virtual biopsy” for predicting these genetic signatures. By extracting quantitative features from manual 3D segmentations of renal tumors in the nephrographic phase (NP) using Logistic Regression (LR model), researchers have reported an AUC of 0.91 in differentiating low-risk from high-risk hypoxia-gene signature expression levels in ccRCC [36]. Random Forest model has also effectively predicted the expression of hypoxia-key biomarkers such as KLF6, ETS1, and BCL2, as well as the underexpression of PLOD2 and PPARGC1A [58]. Texture metrics derived from CT scans, specifically Gray Level Dependence Matrix (GLDM) and Gray Level Co-occurrence Matrix (GLCM), show significant associations with hypoxia biomarkers, maintaining predictive value even across different ccRCC grades and stages [58].

Beyond hypoxia, the metabolic profile of ccRCC is defined by a significant reprogramming of lipid metabolism. A hallmark histological feature of ccRCC is the clear cytoplasm resulting from the massive accumulation of lipids and glycogen. This phenomenon is largely driven by the loss of the VHL tumor suppressor, which causes an accumulation of HIF-α protein. This stabilization of HIF-α suppresses the activity of carnitine palmitoyltransferase 1A, thereby inhibiting the transport of fatty acids into the mitochondria for oxidation. Consequently, fatty acids are diverted into cytoplasmic lipid droplets, providing the essential building blocks for tumor cell survival and proliferation [25,60,61].

The ability to non-invasively assess this lipid-rich landscape has been explored through CT-based radiomics, showing promising, albeit slightly more modest, results compared to hypoxia-gene prediction [25]. Using a signature constructed from nine radiomics features extracted during the NP, researchers reported an AUC of 0.74 for classifying patients into low-risk or high-risk Lipid Metabolism Related Genes expression groups, providing a statistically significant tool for understanding the metabolic heterogeneity of ccRCC and its impact on the tumor’s biological behavior [25]. By integrating both hypoxia and lipid metabolism signatures, radiogenomics offers a comprehensive view of the “pseudo-hypoxic” and lipid-dense environment that characterizes aggressive ccRCC phenotypes.

### 4.3. Biological Processes Markers

In addition to metabolic reprogramming, tumor heterogeneity—driven by the complex interplay between malignant cells and the TME—is a primary determinant of prognostic variability in ccRCC. The TME, composed largely of infiltrating immune and stromal cells, plays a pivotal role in regulating tumor aggressiveness and progression. Specifically, the heterogeneous expression of immune-related genes is closely linked to clinical outcomes, reflecting the tumor’s ability to evade or co-opt the host immune system [34,62,63].

CT radiomics has also proven feasible in characterizing this immunological landscape non-invasively [34]. By analyzing the NP CT data through manual 3D segmentation of the renal tumors, researchers identified 11 key radiomic features capable of predicting the expression of an eight-immune-related gene signature. The resulting radiomics score and radiomics nomogram yielded AUCs of 0.72 and 0.74, respectively [34]. This demonstrates that macroscopic imaging patterns can capture subtle variations in the TME, providing a valuable tool for assessing the immune-related developmental potential of ccRCC and personalizing therapeutic strategies.

Programmed Cell Death (PCD) patterns have emerged as critical determinants of tumorigenesis, clinical prognosis, and therapeutic sensitivity in ccRCC [23,64]. Programmed Cell Death encompasses a complex spectrum of regulated pathways—ranging from classical apoptosis and autophagy to distinct mechanisms such as ferroptosis, necroptosis, and pyroptosis—that maintain homeostasis by eliminating damaged cells. In the context of ccRCC, these diverse modes of cell death are intrinsically linked to the tumor’s ability to survive environmental stress and respond to treatment [23,64].

The clinical relevance of these molecular processes can be captured via CT radiomics, which has demonstrated a robust predictive performance. Utilizing the LR algorithm, radiomic models have achieved an AUC of 0.813 in differentiating between high-risk and low-risk PCD gene pair statuses [23]. Notably, this radiogenomic stratification carries significant therapeutic weight: patients classified as high-risk based on their PCD profiles exhibit increased responsiveness to tyrosine kinase inhibitors (TKIs), mTOR inhibitors, and immunotherapy [23]. This highlights the value of radiomics as a non-invasive tool for predicting not only the biological “death signature” of a tumor but also its potential sensitivity to contemporary systemic therapies.

### 4.4. Molecular Taxonomy Traits

The integration of transcriptomic profiling and quantitative imaging represents another transformative shift in the characterization of ccRCC [35]. By clustering mRNA expression, researchers have identified distinct molecular subtypes (m1–m4) that encapsulate the inherent biological heterogeneity of these tumors and dictate clinical outcomes [35,65,66,67,68,69]. For example, the m1 subtype is characterized by frequent *PBRM1* mutations and significant survival advantages, whereas the m3 and m4 subtypes exhibit more aggressive genetic signatures, including *CDKN2A* deletions, *PTEN* mutations, and a high frequency of *BAP1* and *mTOR* alterations [14]. By utilizing a RF algorithm to analyze shape, histogram, and texture features, researchers have demonstrated exceptional predictive performance, achieving AUC values between 0.953 and 0.973 in predicting mRNA-based molecular subtypes. These findings suggest that the macro-scale phenotypic patterns captured via CT—such as tissue entropy and complexity—directly reflect the underlying micro-scale mRNA landscapes [35].

### 4.5. Targeted Therapeutic Markers

The clinical management of ccRCC is increasingly driven by the identification of specific molecular vulnerabilities, such as mTOR pathway alterations and the Angio RNA expression. The mTOR pathway, closely linked to VHL-induced angiogenesis, serves as a critical therapeutic target; patients harboring gain-of-function mutations in this pathway derive significant clinical benefit from mTOR inhibitors like everolimus [30,70]. Furthermore, the Angio molecular subgroup, defined by a gene signature associated with high vascularity, identifies patients who achieve improved progression-free survival when treated with anti-VEGF monotherapy [30,71]. CT radiomics combined with the AdaBoost algorithm has demonstrated clinical utility in the non-invasive detection of these markers [30]. Research indicates that radiomic models can predict mTOR mutation status with acceptable discrimination (AUC = 0.74–0.77) in advanced stage III/IV and high-grade tumors, while also identifying the Angio RNA expression signature (AUC = 0.71) in grade 3/4 disease [30].

### 4.6. Complex Molecular Traits

A complex genomic landscape in ccRCC, where clinical outcomes are driven by the Weighted Genomic Instability Index (wGII), a measure of chromosomal complexity, and Intratumoural Heterogeneity (ITH), which reflects the diverse genetic sub-clones within a single malignancy was described in the TRACERx Renal study (NCT03226886) [32]. These molecular markers, alongside somatic alterations like the loss of the 9p21.3 marker and the tumor’s Evolutionary Subtype—categorized as linear, branched, or punctuated—provide critical prognostic value but are often inaccessible due to the high cost and logistical constraints of multi-region genomic profiling.

By employing advanced 3D segmentation, both manual and semi-automated, that isolate specific sub-regions—including the tumor core, rim, and areas of high or low enhancement—and by using the LR algorithm researchers achieved significant predictive performance (AUC = 0.736–0.814) for critical metrics such as wGII, ITH index, and loss of 9p21.3 somatic alteration marker [32]. The study further highlights that radiomics features are essential for capturing the biological signatures of aggressive disease, potentially allowing for pre-surgical treatment planning and the longitudinal monitoring of genomic evolution without the need for repetitive, invasive sampling [32].

### 4.7. Tumor Microenvironment

The Tumor Microenvironment in RCC is a highly complex, dynamic ecosystem that serves as a primary driver of therapeutic resistance and disease progression. Its architecture is defined by three critical pillars: intense vascularization, extensive but dysfunctional immune infiltration, and profound metabolic reprogramming [9,72,73,74,75]. Driven by the VHL-HIF axis, RCC exhibits extreme angiogenesis, where the loss of VHL leads to a surge in VEGF and the creation of a disorganized, “leaky” vascular network. Despite being one of the most immune-infiltrated solid tumors, RCC paradoxically utilizes this “immune landscape” to favor the tumor; high levels of CD8+T cells, often correlate with poor outcomes due to T-cell exhaustion mediated by checkpoints like PD-L1 and Cytotoxic T-Lymphocyte Associated Protein 4 (CTLA-4). This immunosuppression is further bolstered by a “harsh” metabolic niche—characterized by hypoxia, acidity, and nutrient competition—where the Warburg Effect and lipid accumulation create an environment that “starves” effector cells while supporting tumor survival. Finally, the structural scaffolding of the TME is actively managed by Cancer-Associated Fibroblasts and a remodeled Extracellular Matrix, which not only facilitate metastasis through increased tissue stiffness but also act as physical and chemical barriers against conventional and targeted therapies [9,72,73,74,75,76].

While treatments like ICIs and targeted therapies have advanced care, clinical responses remain heterogeneous, underscoring the need for a deeper understanding of RCC immunobiology. Current research focuses on identifying TME predictive biomarkers to better stratify patients [76,77].

Quantitative multiplex immunofluorescence (mIF) is a powerful tool for analyzing the TME by simultaneously mapping the spatial distribution and interactions of multiple biomarkers. While macroscopic approaches such as bulk expression profiling or radiomics, often obscure critical biology by averaging features across entire lesions, direct microscopic pathological assessment via mIF preserves tissue architecture. This spatial resolution is vital because the predictive capability of the TME resides predominantly within localized microscopic hotspots—such as functional cell niches or tertiary lymphoid structures—rather than global, macroscopic high or low expression levels. By resolving these intricate architectures, mIF provides critical insights into how cell-to-cell proximity influences disease progression [20,78]. However, mIF limitations in RCC center on intratumoral heterogeneity, where localized tissue sections often fail to represent the entire tumor’s diverse immune landscape. Technical hurdles include renal autofluorescence that obscures signals, and a lack of standardized analytical pipelines for spatial data. Finally, mIF provides only a static snapshot, missing the rapid metabolic and functional shifts characteristic of the RCC microenvironment [78].

#### 4.7.1. CD8+ T Cells/PD-L1/CD68+ Tumor-Associated Macrophages

In RCC, CD8+ T cells (Cluster of Differentiation 8 Positive), often referred to as Cytotoxic T Lymphocytes present a unique clinical paradox: while they are the primary “assassins” of the immune system, their high density within a tumor often correlates with aggressive disease and a poor prognosis. This occurs because the RCC microenvironment frequently drives these cells into a state of functional exhaustion, where they remain present but are rendered inactive by inhibitory signals. These “brakes” are often applied by the surrounding CD68+ Tumor-Associated Macrophages (TAMs) or through the expression of checkpoints like PD-L1 or CDLA4 on tumor cells. Consequently, modern RCC treatments focus on using immunotherapy to “re-awaken” these suppressed CD8+ T cells, transforming them from passive bystanders back into active anti-tumor effectors [9,33,74,75,76,77].

In a retrospective study, a CT-radiomics signature demonstrated moderate accuracy in differentiating ccRCCs with high CD8+ T-cell infiltration (AUC: 0.68; 95% CI: 0.55–0.80) [33]. Histogram, texture, and transformed-based radiomics features were extracted after both 3D manual segmentation of the whole tumor and 2D manual segmentation of the largest tumor diameter. The AdaBoost algorithm had the best performance, and the 3D GLCM proved the optimal discriminator feature, due to the inherent intratumoral heterogeneity of ccRCC [33].

The same study reported a good accuracy for CT-based radiomics in assessing PD-L1 expression levels, overcoming the limitations of traditional biopsy. An Adaboost ML algorithm discriminated between PD-L1 positive and PD-L1 negative ccRCCs with an AUC of 0.8 (95% CI: 0.66, 0.95). The 3D gray level run-length matrix (GLRLM) metrics proved the best discriminating feature, as it captures the broader structural changes in the tumor as a whole [33]. In the ccRCC landscape, PD-L1 acts as a pivotal immune-evasion signal, frequently upregulated by both tumor cells and CD68+ TAMs to deactivate infiltrating CD8+ T cells. While high PD-L1 expression typically correlates with aggressive disease and poor survival, it also serves as a key predictive biomarker for the success of checkpoint inhibitor therapy [33,79,80].

CD68+ TAMs are immune cells of the myeloid lineage that have been recruited into the TME and “re-educated” by cancer cells to support tumor growth rather than destroy it. While CD68 is a general (pan-macrophage) marker used to identify the total presence of these cells, their role in ccRCC is particularly significant as they act as key mediators of angiogenesis, immunosuppression and poor prognosis. In patients with RCC, a high density or spatial clustering of CD68+ TAMs is typically associated with advanced disease stage, high nuclear grade, and worse overall survival [20,27,33,81,82]. These cells facilitate tumor progression by secreting growth factors that promote angiogenesis and by releasing inhibitory cytokines that “exhaust” the CD8+ T cells, effectively creating a shield that prevents the immune system from mounting an effective attack. Within the ccRCC microenvironment, CD68+ TAMs exhibit a diverse range of functional states, primarily divided into two opposing roles. The M1 phenotype acts as a defensive force, promoting anti-tumor activity and aiding in the destruction of cancer cells. Conversely, the M2 phenotype is co-opted by the malignancy to drive tumor growth and help the cancer evade detection by the immune system. Therefore, CD68+ TAMs can become a biomarker, providing information regarding treatment resistance and the likelihood of a patient responding to immunotherapy [20,27,33,81,82].

Preliminary data from two retrospective studies (*n* = 156) report that CT radiomics can feasibly assess CD68+ TAMs noninvasively. Utilizing manual 3D segmentation of the whole renal tumor and a four-phase CT protocol, researchers extracted histogram, texture, and transform-based features to achieve AUCs between 0.77 and 0.85, suggesting strong potential for real-time immune profiling [20,33]. The highest-performing radiomics pipeline, utilizing a Green Learning framework and a Gradient Boosting (XGBoost) algorithm, achieved an AUC of 0.85 (95% CI: 0.76–0.93) for predicting TAM populations [20].

#### 4.7.2. Inflammation-Related Genes

Inflammation-related genes (IRGs) have an important role in the development and progression of ccRCC by shaping the inflammatory TME [39,83,84,85]. Studies have shown that IRGs are closely associated with the infiltration of immune and inflammatory cells, such as activated CD8+ T cells, myeloid-derived suppressor cells, neutrophils, macrophages, and natural killer cells, all of which contribute to tumor progression, angiogenesis, metastasis, and immune evasion. Because ccRCC is a highly immunogenic tumor, the interaction between inflammatory signaling pathways and immune cells strongly influences patient prognosis and survival. Dysregulation of inflammatory mediators and cytokines, including molecules such as MCPIP1, has been linked to poor prognosis and enhanced metastatic potential. Therefore, IRGs are considered promising biomarkers for predicting clinical outcomes and potential therapeutic targets in ccRCC [39,83,84,85].

A radiomics signature built on five features extracted from NP CT data proved reliable in predicting IRGs in ccRCC (AUC: 0.73; 95% CI: 0.60–0.86) [39]. More importantly, IRG-related risk scores showed satisfactory performance in assessing prognosis and improving the management of patients with ccRCC in the same study [39].

#### 4.7.3. CTLA-4

CTLA-4 is a protein receptor expressed on the membranes of activated T-cells that modulates immune responses by downregulating effector T-cells and intensifying regulatory T-cell activity. Although CTLA-4 is significantly overexpressed in RCC tissues—often correlating with an aggressive TME and poor prognosis—this overexpression simultaneously identifies a ‘primed’ target for dual-checkpoint inhibition [29,86,87]. CTLA-4 mRNA expression in ccRCC can be predicted noninvasively using a radiomics model based on NP CECT. A Support Vector Machine (SVM) algorithm utilizing seven radiomics features achieved an AUC of 0.724 (95% CI: 0.613–0.835). Furthermore, a radiomics nomogram integrating the radiomics signature with clinicopathologic factors predicted overall survival with high efficiency, reaching an AUC of 0.826 at 12 months [29].

#### 4.7.4. FOXP3/FOXM1

FOXP3 (forkhead box protein 3) serves as the master transcription factor for regulatory T-cells (Tregs) and is a critical mediator of immune evasion in RCC. High intratumoral FOXP3+ expression is consistently associated with advanced tumor stage and serves as a robust negative prognostic biomarker. Furthermore, the FOXP3+ cell density within the TME modulates the efficacy of dual-checkpoint inhibition, marking it as a focal point for future T-cell-directed therapies [24,88]. Determining FOXP3 status in RCC involves several methodologies, each with distinct technical limitations [24,88,89].

Utilizing a Gradient Boosting Machine (GBM) algorithm, researchers developed a non-invasive radiomics model based on three CT-derived radiomics features that effectively predicts FOXP3 expression levels, achieving a commendable AUC of 0.835 [24]. The same study demonstrates that FOXP3 serves as an independent prognostic marker for ccRCC, with high expression significantly correlating with decreased overall survival [24].

Another member of the Forkhead Box family of transcription factors is the Forkhead box M1(FOXM1), serving as a master regulator in the pathogenesis of ccRCC [90]. Structurally characterized by its N-terminal transactivation, central DNA-binding, and C-terminal protein-interaction domains, FOXM1 drives malignancy by modulating the transcription of genes essential for cell cycle progression (such as cyclin B1 and D1), DNA repair, and the epithelial–mesenchymal transition pathway. Furthermore, it facilitates tumor vascularization by upregulating pro-angiogenic factors like VEGF and matrix metalloproteinases. Clinically, elevated FOXM1 levels are robustly correlated with advanced TNM stages and poor overall survival, identifying it as a critical biomarker for risk stratification [91,92,93].

A CT-based radiomics signature proved a reliable, non-invasive predictor of FOXM1 expression and a robust prognostic tool for patients with ccRCC. By analyzing radiomics features from the NP in 184 patients, researchers developed a model that achieved solid predictive accuracy (AUC = 0.711) for identifying high FOXM1 expression. Crucially, when the Rad-score was integrated with traditional clinical factors and TNM staging into a combined model, it yielded the highest prognostic performance [28].

### 4.8. Ki67 Proliferation

Ki-67 is a nuclear antigen expressed during all active phases of the cell cycle—G1, S, G2, and mitosis (M)—and is notably absent during the resting phase (G0). As this molecular marker increases incrementally from G1 to M and declines rapidly thereafter, it is used to quantify the “growth fraction” of a cell population and is closely related to the tumor cells’ proliferation and invasion [18,19,21,22,26,31]. In RCC, Ki-67 acts as a critical prognostic biomarker; high expression levels are strongly correlated with aggressive tumor behavior, poor prognosis, and adverse clinicopathological features, potentially serving as a marker for risk stratification or even a therapeutic target. For patients with highly proliferative tumors based on their Ki-67 index, close postoperative monitoring, adjunctive immunotherapy, and targeted therapy are recommended [18,19,21,22,26,31,94,95].

The evaluation of Ki-67 is currently performed through standard IHC. However, this technique is invasive and cannot be used for continuous dynamic monitoring during patient follow-up. In addition, when obtained through biopsy material, it may fail to represent the entire tumor due to intratumoral heterogeneity [96,97].

Based on the results of six retrospective multicenter studies (*n* = 4.271), CT-based radiomics has shown strong to excellent predictive capability for assessing Ki-67 expression levels in RCC. Artificial Intelligence models proved robust across different institutions, with AUCs ranging between 0.668 and 0.885 [18,19,21,22,26,31]. Five studies reported the CECT protocol used: one study used a four-phase CT protocol [18], one study utilized the CMP [19], and three studies used two phases (CMP and NP) [21,22,26]. Segmentation techniques included manual 3D [18,21,26] and automated 3D [19,22,31] segmentation of the whole tumor. While various ML algorithms were employed, texture features [21] were most frequently used, often in combination with shape and histogram features [19,22,31] or transform-based features [18,26].

The highest diagnostic performance was reported in a study of 506 patients with ccRCC [18]. Ki-67 expression was assessed by IHC and categorized into low- (<15%) and high-expression (≥15%) groups. Manual 3D segmentation of the tumor was performed on CMP CT images, and a total of 1316 radiomics features were extracted. Eight radiomics features were selected to train five ML models: RF, XGBoost, LR, SVM, and K-Nearest Neighbor (kNN). The RF model exhibited the best performance, with an AUC of 0.885 in the external test set [18]. The same study also reported that both the IHC-confirmed and RF-predicted high Ki-67 expression groups were significantly associated with a higher risk of tumor recurrence [18].

Table 4 presents an overview of the methodological strategies reported in the reviewed literature—spanning segmentation, feature extraction, harmonization, ML algorithms, validation, and software reporting—alongside current recommendations to improve reproducibility and generalizability.

### 4.9. Limitations

This review has several limitations that should be acknowledged. First, all included studies had a retrospective design, which increases the risk of selection bias and limits the overall level of evidence. In addition, many studies were based on publicly available datasets, such as TCGA-TCIA and TCGA-KIRC, potentially resulting in overlapping patient populations and reduced generalizability of findings. To demonstrate true clinical utility, future models must move beyond these standard public repositories and undergo validation on multi-institutional datasets that reflect modern, real-world scanning parameters.

Second, substantial methodological heterogeneity was observed among the included studies. Differences in CT acquisition phases, segmentation techniques, radiomics feature extraction methods, and ML algorithms limited direct comparison between studies and precluded quantitative synthesis or meta-analysis. Furthermore, the lack of standardized radiomics workflows remains a major challenge for reproducibility and clinical translation.

Regarding tumor segmentation strategies, manual whole-tumor segmentation was the predominant method used in the reviewed studies. However, this technique is time-consuming, labor-intensive, and prone to intra- and inter-observer variability. To overcome these limitations and standardize the radiogenomic workflow, emerging pipelines are increasingly adopting semi-automated and deep learning-based, fully automated segmentation methods. Semi-automated methods reduce processing time by using algorithm-generated boundaries that can be refined by experts, while fully automated deep-learning approaches provide rapid, reproducible, and standardized region-of-interest delineation without observer bias. By enabling consistent, user-independent tumor segmentation, these approaches improve workflow efficiency and facilitate the clinical translation of radiogenomic models.

Another important limitation of this review was the inconsistent use of validation strategies. Several studies relied exclusively on internal validation or cross-validation without independent external validation cohorts, increasing the likelihood of model overfitting and overestimation of predictive performance. In addition, reporting of model performance metrics and radiomics methodologies was incomplete in some studies, further limiting reproducibility.

This review also focused exclusively on CT-based radiogenomics in patients with ccRCC, excluding other RCC histological subtypes and imaging modalities such as magnetic resonance imaging and positron emission tomography. Consequently, the findings may not be generalizable to the broader RCC population.

Another potential optimism bias should be acknowledged, particularly because several studies reported multiple biomarkers, ML models, or validation strategies, and the available performance metrics may preferentially reflect the best-performing results. To reduce overrepresentation of studies reporting multiple endpoints, a conservative study-level descriptive summary was provided; however, this approach does not eliminate the risk of selective reporting. Because this was a comprehensive literature review and the included studies were highly heterogeneous, the study-level AUC summary should be interpreted only as a descriptive aid and not as a pooled estimate of model performance. Therefore, the findings of our review should be interpreted as a descriptive overview of the currently available literature rather than definitive evidence supporting immediate clinical implementation.

It is also critical to acknowledge that the independent prognostic value of molecular markers may be overestimated in the current literature. From a clinicopathological standpoint, when analyses are restricted to stage-matched cohorts or when confounding anatomical parameters are rigorously controlled, the standalone impact of these molecular shifts on patient survival is often limited. The TNM staging system remains the most robust and clinically validated prognostic determinant in ccRCC. While radiogenomic signatures aim to capture microscopic intratumoral heterogeneity, they must be viewed as tools to refine, rather than supplant, established staging guidelines. Readers should exercise caution when interpreting the clinical predictive weight of individual molecular markers outside the context of anatomical tumor staging.

Finally, in clinical settings characterized by low-volume disease—such as early-stage sub-centimeter tumors, or minimal residual and oligometastatic disease—CT-based radiomics encounters severe physical and technical constraints. The limited spatial resolution of cross-sectional imaging induces significant partial volume effects, making precise structural delineation and high-order texture feature extraction mathematically unreliable. In contrast, micro-scale tissue sampling methods, including core needle biopsy and ultra-sensitive genomic sequencing (e.g., next-generation sequencing or liquid biopsies), can directly isolate and identify low-abundance cellular and genetic alterations long before they manifest as identifiable macroscopic imaging phenotypes.

## 5. Conclusions and Future Directions

CT-based radiogenomics has emerged as a promising non-invasive approach for the molecular characterization of ccRCC. Current evidence demonstrates that quantitative radiomics features extracted from routine contrast-enhanced CT can predict a broad spectrum of molecular and biological biomarkers, including gene mutations, hypoxia-related pathways, immune-related signatures, proliferation markers, and tumor microenvironment characteristics. The integration of ML algorithms has further improved predictive performance, with several studies reporting high diagnostic accuracy for clinically relevant biomarkers such as VHL, PBRM1, BAP1, PD-L1, and Ki-67. In a standardized clinical workflow, this begins with routine CECT acquisition, followed by tumor segmentation, feature extraction, and ML-driven feature selection to generate a patient-specific radiomic risk signature. Within a clinical trial context, such a radiomics signature could fundamentally alter patient management by serving as a non-invasive, spatial enrichment tool. For instance, rather than relying on a single, static needle biopsy that is highly vulnerable to intratumoral heterogeneity, a radiomics signature predicting a high-risk *BAP1* mutation or high PD-L1 expression could be used at baseline to stratify patients into targeted TKI versus combination ICI cohorts. Furthermore, during a trial, tracking feature changes over time could alter management by identifying a rapid shift toward an aggressive, immunosuppressed phenotype weeks before macroscopic RECIST criteria register disease progression. This would allow investigators to safely cross-over non-responders to secondary treatment arms or modify therapeutic dosages early, drastically reducing toxicity and optimizing precision trial designs. These findings highlight the potential of radiogenomics to support personalized treatment strategies, prognostic stratification, and non-invasive longitudinal monitoring in patients with ccRCC.

However, despite these encouraging results, significant limitations currently hinder translation into routine clinical practice. Most available studies are retrospective, single-center investigations with relatively small and heterogeneous cohorts. Considerable variability exists in CT acquisition protocols, segmentation methods, radiomics workflows, and ML models, limiting reproducibility and external generalizability. Furthermore, many studies rely solely on internal validation, increasing the risk of overestimating model performance. Therefore, current evidence should be interpreted cautiously.

Future research must focus on the development of large prospective, multicenter studies utilizing harmonized radiomic pipelines and standardized acquisition protocols to ensure robust external validation across diverse patient populations. Crucially, the future of this field hinges on integrating advanced Artificial Intelligence (AI)—particularly deep learning and automated segmentation techniques—to eliminate inter-observer dependency and facilitate fully automated radiogenomic workflows viable for real-world clinical practice. Furthermore, transitioning from static, single-time-point assessments to longitudinal imaging analysis will allow clinicians to dynamically track tumor evolution, monitor early treatment response, and detect resistance mechanisms during targeted therapy or immunotherapy. Ultimately, by combining these automated pipelines with genomics, transcriptomics, and pathomics within a comprehensive multi-omics framework, CT-based radiogenomics has the potential to evolve into a powerful, non-invasive virtual biopsy tool that supports precision oncology through individualized risk assessment and optimized treatment selection in ccRCC.

## Figures and Tables

**Table 1:** Baseline characteristics of the studies included in the review for predicting molecular markers in patients with clear cell renal cell carcinoma.

Study	Year	Outcome	No of Patients with Renal Tumors	CT Phase
Xv et al. [18]	2025	to predict Ki-67 expression	506	UECT, CMP, NP, EP
Yang et al. [19]	2025	to predict Ki-67 expression	1073	CMP
Wu et al. [20]	2025	to predict CD68 TAMs	78 *	UECT, CMP, NP, EP
Li et al. [21]	2025	to predict Ki-67 expression	627	CMP, NP
Yang et al. [22]	2024	to predict Ki-67 expression	1051	CMP, NP
Chen et al. [23]	2024	to predict CDRGPS	267	n/a
Wang et al. [24]	2024	to predict FOXP3 expression	430	n/a
He et al. [25]	2024	to predict lipid metabolism-related genes (*n* = 13)	182	n/a
Li et al. [26]	2024	to predict Ki-67 expression	185	CMP, NP
Shieh et al. [27]	2024	to predict CD68 TAMs: TAM population + tumor-TAM clustering	78 *	UECT, CMP, NP, EP
Zhao et al. [28]	2024	to predict FOXM1 expression	529	NP
He et al. [29]	2023	to predict CTLA4 expression levels	102	NP
Liu et al. [30]	2023	to predict molecular biomarkers (*n* = 12)	190	UECT, CMP, NP, EP
Yang et al. [31]	2023	to predict Ki-67 expression	588	n/a
Orton et al. [32]	2023	to predict molecular biomarkers (*n* = 14)	86 *	NP
Varghese et al. [33]	2022	to predict CD8-T cell infiltration + PD-L1 expression	78 *	CMP, NP, EP
Gao et al. [34]	2022	to predict immune-related genes (*n* = 8)	193	CMP, NP, EP
Zeng et al. [35]	2021	to predict genetic mutations + mRNA-based molecular subtypes	382	n/a
Gao et al. [36]	2021	to predict hypoxia-genes: IFT57, PABPN1, RNF10, RNF19B, UBE2T	194	UECT, CMP, NP
Feng et al. [37]	2020	to predict BAP1 mutation status	54 *	NP
Kocak et al. [38]	2020	to predict BAP1 mutation status	65 *	UECT
Yang et al. [39]	2023	to predict inflammation-related genes	571	NP
Kocak et al. [40]	2018	to predict PBRM1 mutation status	45 *	CMP
Chen et al. [41]	2018	to predict VHL, PBRM1, and BAP1 gene mutations	57 *	CMP
Ghosh et al. [42]	2015	to predict BAP1 mutation status	14 *	UECT, CMP, NP, EP

No: number; BAP1: Breast Cancer gene 1-associated protein 1; UECT: unenhanced computed tomography; CMP: corticomedullary phase; NP: nephrographic phase; EP: excretory phase; VHL: Von Hippel–Lindau; PBRM1: Polybromo 1; mRNA: messenger ribonucleic acid; n/a: not available; FOXM1: Forkhead Box M1; CTLA-4: Cytotoxic T-Lymphocyte–Associated Protein 4; FOXP3: Forkhead Box P3; CD8+ T cell: Cluster of Differentiation 8–positive T lymphocyte; PD-L1: programmed death-ligand 1; CD68: Cluster of Differentiation 68; TAMs: tumor-associated macrophages; CDRGPS: cell death-related gene pair score. * Studies with fewer than 100 patients are indicated with an asterisk.

**Table 2:** Characteristics of the radiomics pipelines used in studies included in the review for predicting molecular markers in patients with clear cell renal cell carcinoma.

Study	Segmentation Method	Extracted Features	Model Training	AUC (±SD OR 95% CI)
Xv et al. [18]	manual 3D, whole volume, CMP	n/a	RF	0.885 (0.826–0.934)
Yang et al. [19]	automated, 3D UNet, whole volume	100 features: first-order, shape, texture	XGBoost	0.82 (0.74–0.88)
Wu et al. [20]	manual 3D, whole volume	1708 texture features: 3D intensity/ histogram, 2D + 3D GLSZM, 2D + 3D LTE, 2D + 3D GLRLM, 2D FFT, 3D GLDM, 2D DCT, 2D + 3D GLCM	XGBoost	0.85 (0.76–0.93)
Li et al. [21]	manual 3D, whole volume	1834 features: conventional (first-order, shape, texture + filtered), + Habitat + peritumoral 1 mm, 3 mm, 5 mm	ExtraTrees	0.742 (0.676–0.808)
Yang et al. [22]	automated 3D Unet kidneys + kidney tumor segmentation	200 features/phase	XGBoost	0.81 (0.74–0.89)
Chen et al. [23]	manual 3D, whole volume	1688 features	LASSO	0.813
Wang et al. [24]	manual 3D, whole volume	107 features	GBM	0.809
He et al. [25]	manual 3D, whole volume, NP	1316 features: shape, first-order, GLDM, GLCM, GLRLM, GLSZM, NGTDM, wavelet	LASSO	0.74
Li et al. [26]	manual 3D, whole volume	3376 features: intensity statistics, shape, texture (GLDM, GLCM, GLRLM, GLSZM, NGTDM), filter and wavelet	LASSO	clinical model: 0.698 (0.546–0.849)
				radiomics signature: 0.799 (0.668–0.929)
				radiomics nomogram: 0.814 (0.704–0.925)
Shieh et al. [27]	manual 3D, whole volume	438 texture features: 3D intensity/ histogram, 2D + 3D GLSZM, 2D + 3D LTE, 2D + 3D GLRLM, 2D FFT, 3D GLDM, 2D DCT, 2D + 3D GLCM	RF	TAM population: 0.81 (0.69–0.92)
			AdaBoost	TAM clustering: 0.77 (0.66–0.88)
Zhao et al. [28]	manual 3D, whole volume	107 features	LASSO	0.711
He et al. [29]	manual 3D, whole volume	107 texture features: first-order, shape, GLRLM, GLCM, GLDM, GLSZM, NGTDM	SVM	0.724 (0.613–0.835)
Liu et al. [30]	manual 3D, whole volume + 2D, maximum tumor diameter	2.824 texture features	AdaBoost	KDM5C (stage I/II): 0.77
			RF	SETD2 (stage I/II): 0.86
			RF	PBRM1 (stage III/IV): 0.77
			AdaBoost	Angio RNA expression (grade 3/4): 0.71
			AdaBoost	mTOR (stage III/IV): 0.77
Yang et al. [31]	automated, 3D Unet kidneys + kidney tumor segmentation	100 features: texture, morphologic, statistical	XGBoost	0.82 ± 0.1 (0.74–0.91)
Orton et al. [32]	manual 3D, whole volume + semiautomated: core, rim, high and low-enhancing subregions	105 features: shape, first-order, texture	LASSO	ITH index: 0.745 wGII Max: 0.737 Loss9p21.3: 0.814
Varghese et al. [33]	manual 3D, whole volume + 2D, maximum tumor diameter	2824 features: 3D Intensity/Histogram, 2D + 2D GLSZM, 2D + 3D LTE, 2D + 3D GLRLM, 2D FFT, 2D + 3D GLDM,2D DCT, 2D + 3D GLCM	RF, AdaBoost, Elastic Net	CD8-T cell infiltration: 0.68 (0.55–0.80)
				PD-L1: 0.80 (0.66–0.95)
Gao et al. [34]	manual 3D, whole volume, NP	1218 features: shape, first-order, GLCM, GLRLM, GLSZM, GLDM, wavelet + LoG transforms	LASSO	0.91
Zeng et al. [35]	manual 3D, whole volume	107 features: shape-based, histogram-based, GLCM, GLDM, GLRLM, GLSZM, NGTDM	RF	VHL: 0.971
				BAP1: 0.955
				PBRM1: 0.972
				SETD2: 0.949
				mRNA (m1): 0.973
				mRNA (m2): 0.968 mRNA (m3): 0.961
				mRNA (m4): 0.953
Gao et al. [36]	manual 3D, whole volume	1218 features: shape, first-order, GLCM, GLRLM, GLSZM, GLDM (NP)	LASSO	0.91
Feng et al. [37]	manual 2D, maximal tumor slice (axial) + upper, lower slices (3–4 slices skipping)	texture features: intensity histogram, intensity direct, GLCM, NGTDM, GLRLM, original + filtered (LoG)	RF	0.77 (0.70–0.83)
Kocak et al. [38]	manual 2D, 1–4 slices, including maximum tumor diameter	744 texture features: first-order, GLDM, GLCM, GLRLM, GLSZM, NGTDM, wavelet	RF	0.897
Yang et al. [39]	manual 3D, whole volume	1218 features	LASSO	clinical model: 0.52 (0.38–0.66)
				radiomics signature: 0.73 (0.60–0.86)
				radiomics nomogram: 0.71 (0.57–0.84)
Kocak et al. [40]	manual, 3–5 slices, including maximum tumor diameter, 2 mm shrinkage	828 texture features: first-order, GLDM, GLCM, GLRLM, GLSZM, NGTDM, wavelet-based	RF	0.987
Chen et al. [41]	manual 3D, whole volume	43 features: shape, intensity, texture	RF	VHL: 0.88 ± 0.01
			RF	PBRM1: 0.86 ± 0.02
			RF	BAP1: 0.93 ± 0.02
Ghosh et al. [42]	manual 3D	73,684 original + filtered features/phase: intensity + texture	RF	0.71

AUC: area under the curve; SD: standard deviation; CI: confidence interval; 3D: Three dimentional; RF: random forest; VHL: Von Hippel–Lindau; PBRM1: Polybromo 1; BAP1: Breast Cancer gene 1-associated protein 1; GLDM: gray-level dependence matrix; GLCM: gray-level co-occurrence matrix; GLRLM: gray-level run length matrix; GLSZM: gray-level size zone matrix; NGTDM: neighboring gray-tone difference matrix; LoG: Laplacian of Gaussian; 2D: Two dimentional; NP: nephrographic phase; SETD2: SET domain containing 2; mRNA: messenger ribonucleic acid; LASSO: least absolute shrinkage and selection operator; LTE: local texture estimator; FFT: fast Fourier transform; DCT: discrete cosine transform; AdaBoost: adaptive boosting; Elastic Net: elastic net regularization; PD-L1: programmed death-ligand 1; 3D U-Net: three-dimensional U-Net; XGBoost: extreme gradient boosting; KDM5C: lysine demethylase 5C; mTOR: mammalian target of rapamycin; SVM: support vector machine; GBM: gradient boosting machine; CMP: corticomedullary phase; ITH: Intratumoral Heterogeneity; wGII: Weighted Genomic Instability Index.

**Table 3:** Conservative study-level summary of radiomics signature performance by biomarker category.

Molecular Markers	Studies Included	AUC, Median (Range)
Gene mutations	Liu et al. [30]; Zeng et al. [35]; Feng et al. [37]; Kocak et al. [38]; Kocak et al. [40]; Chen et al. [41]; Ghosh et al. [42]	0.880 (0.710–0.987)
Downstream metabolic and biological pathways	He et al. [25]; Gao et al. [36]	0.825 (0.740–0.910)
Biological process markers	Chen et al. [23]; Liu et al. [30]; Gao et al. [34]	0.813 (0.770–0.910)
Molecular taxonomy traits	Zeng et al. [35]	0.964 (0.964–0.964)
Targeted therapeutic markers	Liu et al. [30]	0.710 (0.710–0.710)
Complex molecular traits	Orton et al. [32]	0.745 (0.745–0.745)
Tumor microenvironment	Wu et al. [20]; Wang et al. [24]; Shieh et al. [27]; Zhao et al. [28]; He et al. [29]; Varghese et al. [33]; Yang et al. [39]	0.740 (0.711–0.850)
Ki-67	Xv et al. [18]; Yang et al. [19]; Li et al. [21]; Yang et al. [22]; Li et al. [26]; Yang et al. [31]	0.815 (0.742–0.885)

**Table 4:** Summary of radiomics pipeline components, predominant approaches, and best practices across the reviewed studies.

Pipeline Component	Predominant Approaches Reported in the Reviewed Studies	Clinical Considerations/ Best Practices
**Segmentation**	• manual 3D whole-tumor segmentation (predominant approach) • manual 2D segmentation in earlier studies • automated or semi-automated 3D segmentation using deep-learning approaches (e.g., 3D U-Net) in recent studies	Whole-tumor 3D segmentation better captures intratumoral heterogeneity than 2D approaches. Automated segmentation may improve reproducibility and reduce observer variability, although further validation is needed
**Radiomics Features extracted**	• first-order intensity features • shape features • texture features (GLCM, GLRLM, GLSZM, GLDM, NGTDM) • transform-based features (Wavelet, Laplacian of Gaussian)	Comprehensive feature extraction improves characterization of tumor heterogeneity. Standardized feature definitions (e.g., IBSI recommendations) are desirable to facilitate comparison between studies
**Feature Harmonization**	• harmonization methods were rarely reported • a few studies described intensity normalization or voxel resampling • scanner harmonization methods (e.g., ComBat) were generally not reported	Harmonization is particularly important for multicenter datasets to reduce scanner-related variability while preserving biologically relevant information. Future studies should explicitly report harmonization strategies
**Feature Selection and ML** **algorithms**	**Feature selection:** LASSO, RF, Elastic Net, statistical analysis **Classification algorithms:** RF, XGBoost, AdaBoost, LR, SVM, Gradient Boosting, ExtraTrees	Tree-based ensemble methods (particularly RF and XGBoost) were among the most frequently used algorithms and generally demonstrated robust predictive performance Appropriate feature selection and external validation remain essential to reduce overfitting
**Model Validation**	• Internal validation (cross-validation or hold-out testing) predominated • External validation was performed in 11 out of 25 studies	External validation is critical for assessing model generalizability and should become standard practice before clinical implementation
**Software Reporting**	Software packages used for segmentation, radiomics extraction, or ML were inconsistently reported across studies	Future radiomics studies should explicitly report software packages (e.g., segmentation platform, feature extraction software, ML libraries), software versions, preprocessing settings, and adherence to reporting guidelines to improve reproducibility

3D: three-dimensional; 2D: two-dimensional; GLCM: Gray-Level Co-occurrence Matrix; GLRLM: Gray-Level Run Length Matrix; GLSZM: Gray-Level Size Zone Matrix; GLDM: Gray-Level Dependence Matrix; NGTDM: Neighboring Gray-Tone Difference Matrix; IBSI: Image Biomarker Standardization Initiative; ComBat: Combat harmonization method for batch-effect correction; LASSO: Least Absolute Shrinkage and Selection Operator; RF: Random Forest; XGBoost: Extreme Gradient Boosting; AdaBoost: Adaptive Boosting; LR: Logistic Regression; SVM: Support Vector Machine.

## Data Availability

No new data were created or analyzed in this study. Data sharing is not applicable to this article.

## Abbreviations

2D	Two-Dimensional
3D	Three-Dimensional
AI	Artificial Intelligence
AdaBoost	Adaptive Boosting
AKT	Protein Kinase B
ANN	Artificial Neural Network
Anti-PD1	Anti-Programmed Cell Death Protein 1
AUC	Area Under the Curve
BAP1	BRCA1-Associated Protein 1
ccRCC	Clear Cell Renal Cell Carcinoma
CDRGPS	Cell Death-Related Gene Pair Score
CECT	Contrast-Enhanced Computed Tomography
CD8+	Cluster of Differentiation 8 Positive
CI	Confidence Interval
CMP	Corticomedullary Phase
CTLA-4	Cytotoxic T-Lymphocyte Associated Protein 4
FOXM1	Forkhead Box M1
FOXP3	Forkhead Box Protein 3
GBM	Gradient Boosting Machine
GLCM	Gray Level Co-occurrence Matrix
GLDM	Gray Level Dependence Matrix
GLRLM	Gray Level Run-Length Matrix
GLUT1	Glucose Transporter 1
HIF	Hypoxia-Inducible Factor
ICIs	Immune Checkpoint Inhibitors
IHC	Immunohistochemistry
IRGs	Inflammation-related genes
ITH	Intratumoral Heterogeneity
KDM5C	Lysine Demethylase 5C
kNN	K-Nearest Neighbor
LR	Logistic Regression
MeSH	Medical Subject Headings
mIF	Multiplex Immunofluorescence
ML	Machine Learning
mTOR	Mammalian Target of Rapamycin
NP	Nephrographic Phase
OS	Overall Survival
PBRM1	Polybromo 1
PCD	Programmed Cell Death
PD-L1	Programmed Death-Ligand 1
PFS	Progression-Free Survival
PI3Kβ	Phosphoinositide 3-Kinase Beta
RCC	Renal Cell Carcinoma
RF	Random Forest
RFS	Recurrence-Free Survival
SETD2	SET Domain-Containing 2
SVM	Support Vector Machine
SWI/SNF	SWItch/Sucrose Non-Fermentable
TAMs	Tumor-Associated Macrophages
TCGA-KIRC	The Cancer Genome Atlas Kidney Renal Clear Cell Carcinoma
TCIA	The Cancer Imaging Archive
TKIs	Tyrosine Kinase Inhibitors
TME	Tumor Microenvironment
TNM	Tumor, Node, Metastasis
Tregs	Regulatory T Cells
UECT	Unenhanced Computed Tomography
VEGF	Vascular Endothelial Growth Factor
VHL	Von Hippel–Lindau
wGII	Weighted Genomic Instability Index
XGBoost	Extreme Gradient Boosting
